# Supplementary material for: Intellectual disability-associated factor Zbtb11 cooperates with NRF-2/GABP to control mitochondrial function
Source: Nat Commun. 2020 Oct 29;11:5469. doi: 10.1038/s41467-020-19205-x (PMC7596099; doi:10.1038/s41467-020-19205-x)
Supplement: Supplementary file 1 — Supplementary Figures [file 41467_2020_19205_MOESM1_ESM.pdf]

Intellectual disability-associated factor Zbtb11 cooperates with NRF-2/GABP to control mitochondrial function

Supplementary Information

Wilson B et al

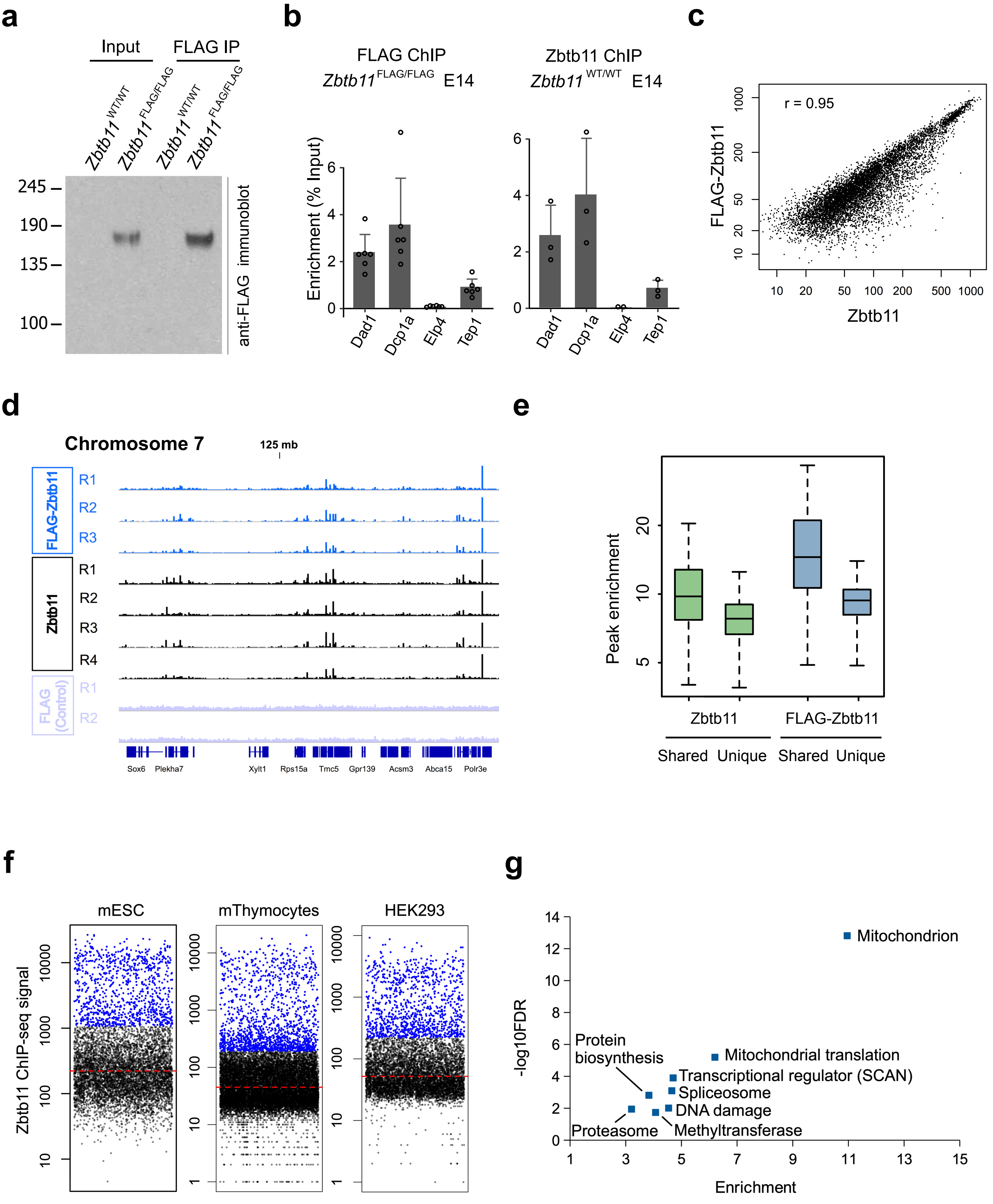

Supplementary Fig. 1

Supplementary Fig. 1.

(a) Immunoblot with anti-FLAG antibodies showing specific and efficient immunoprecipitation (IP) of FLAG-Zbtb11 from chromatin lysates (Input) of *Zbtb11*$^{\mathrm{FLAG/FLAG}}$ E14 cells but not from lysates of the wild type parental line. Molecular weight marker unit is KDa. Source data are provided as a Source Data file.

(b) qPCR analysis of FLAG ChIP samples from *Zbtb11*$^{\mathrm{FLAG/FLAG}}$ E14 cells and of anti-Zbtb11 ChIP from the parental wild type E14 line. Bars show mean and SD of ChIP enrichment values (as percentage of input) obtained from three biological replicates. The panel of primers amplify the promoter regions at the indicated genes. Note the similar distribution of enrichment values obtained with the two different approaches. Source data are provided as a Source Data file.

(c) Correlation of Zbtb11 and FLAG-Zbtb11 ChIP-seq signal at consensus peaks. Plotted values represent mean normalised coverage obtained by averaging the individual biological replicates from each dataset. Pearson's r is shown.

(d) ChIP-seq signal across part of chromosome 7 illustrating the reproducible enrichment obtained for both FLAG-Zbtb11 ChIP in *Zbtb11*$^{\mathrm{FLAG/FLAG}}$ E14 cells, and Zbtb11 ChIP in the parental wild type E14 line. By contrast, no enrichment was generated by the control ChIP experiment using FLAG antibodies in the parental E14 line**.**

(e) Box plot showing the distributions of FLAG-Zbtb11 and Zbtb11 ChIP enrichment values (as obtained during peak calling, see Methods) at peaks that are common or unique to the two datasets. Note that peaks detected with only one of the two approaches are significantly weaker than the common peaks, which makes them more likely to be false positives. Box plots show the interquartile range (box outline) and median value (horizontal line), with the whiskers delineating the lower and upper limits of the data.

(f) Distribution of Zbtb11 ChIP signal in mESC, mouse thymocytes and HEK293 cells. Outlier values (1.5x the interquartile range above the third quartile) used to designate Zbtb11-high peaks are shown in blue. The median of the distribution is shown by the red dashed line.

(g) DAVID functional enrichment analysis of genes with Zbtb11-high peaks at their promoters in human HEK293 cells. Plot shows enrichment values vs significance (-$\log_{10}$FDR) when compared to all genes in the human genome.

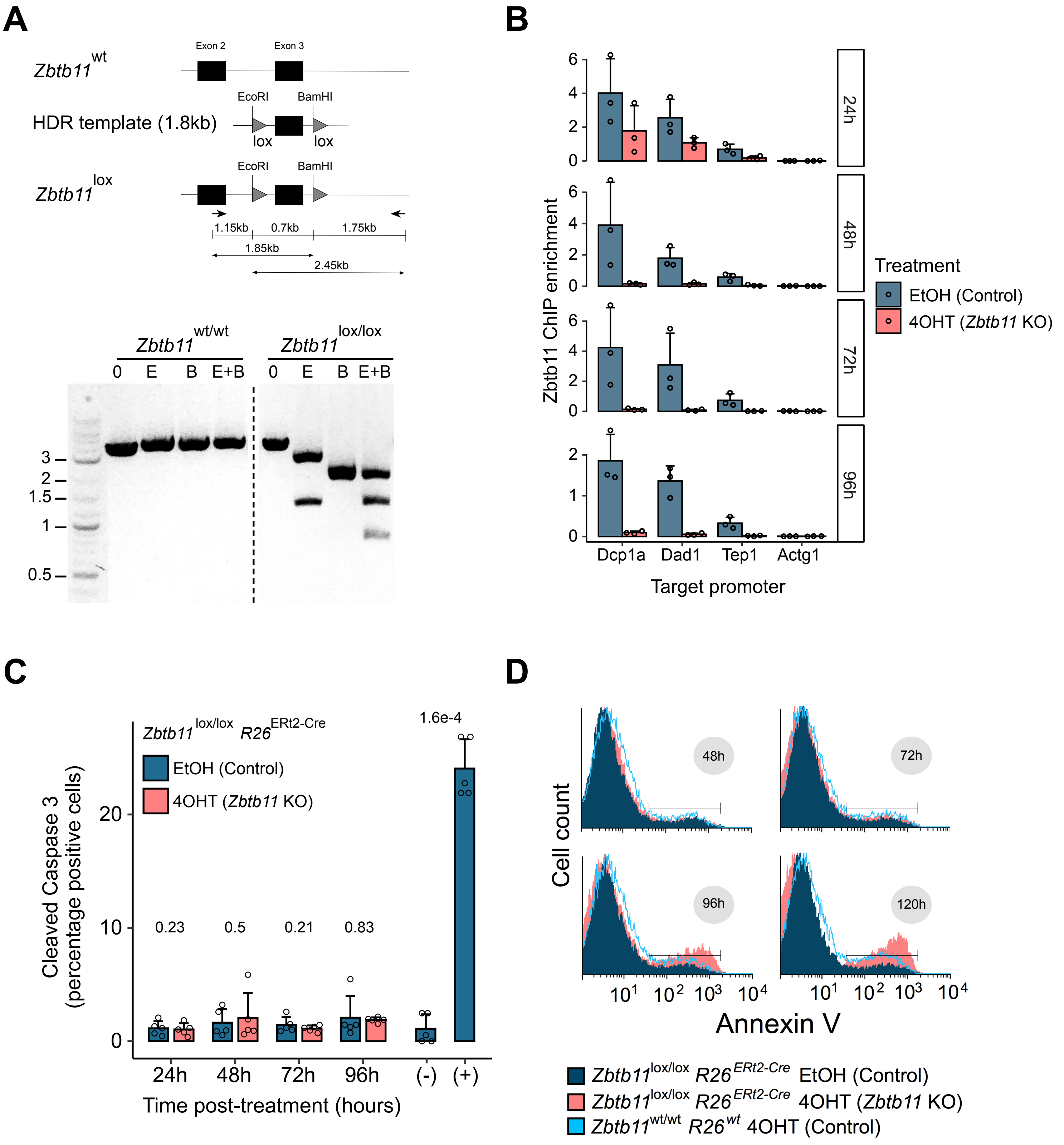

Supplementary Fig. 2

Supplementary Fig. 2

(a) Restriction enzyme digest analysis of the *Zbtb11* locus in *Zbtb11*$^{lox/lox}$ *Rosa26*$^{ERt2\text{-}Cre}$ ESC line. Successful insertion of the two loxP sites flanking exon 3 introduces an ectopic EcoRI site in intron 2 and a BamHI site in intron 3. A 3.6kb region spanning intron 2, exon 3 and part of intron 3 was amplified from gDNA using primers outside the region of homology with the donor template (HDR). Lower panel - DNA agarose gel showing that the PCR amplicon from the parental wild type line is not digested by either EcoRI or BamHI (left), while the amplicons from the recombinant homozygous line are digested by both EcoRI and BamHI (right). The dotted vertical line in the lower panel indicates where intervening lanes have been removed from the image. The unit of the size marker is kb.

(b) ChIP-qPCR with anti-Zbtb11 antibodies in *Zbtb11*$^{lox/lox}$ *Rosa26*$^{ERt2\text{-}Cre}$ cells treated with either EtOH (control) or 4OHT (*Zbtb11* KO) (treatment duration indicated on the right). The primers amplify promoter regions at the indicated genes. Bars show mean and SD of three biological replicates. Note the complete depletion of Zbtb11 protein 48 hours after KO is induced.

(c) Quantification of apoptotic cells following *Zbtb11* KO. *Zbtb11*$^{lox/lox}$ *Rosa26*$^{ERt2\text{-}Cre}$ cells were treated with either EtOH or 4OHT and samples were subsequently collected and fixed at 24 hour intervals. Cells were then stained with fluorescently labelled antibodies to Caspase-3 and analysed by flow cytometry to measure the fraction of Caspase-3 positive cells. Bars show mean and SD of 5 biological replicates, with two-tailed t-test p-values above. Note that this approach did not reveal an increase in apoptotic cells despite being able to specifically and efficiently detect apoptosis in a positive control sample made up of cells submitted to heat-shock (+).

(d) Representative histograms showing the distribution of Annexin V-binding cells as measured by flow cytometry, which was used as a marker of cell death. Line interval demarcates Annexin V-positive cells. Note that an increase in Annexin V-binding cells becomes evident 96 hours after *Zbtb11* KO induction.

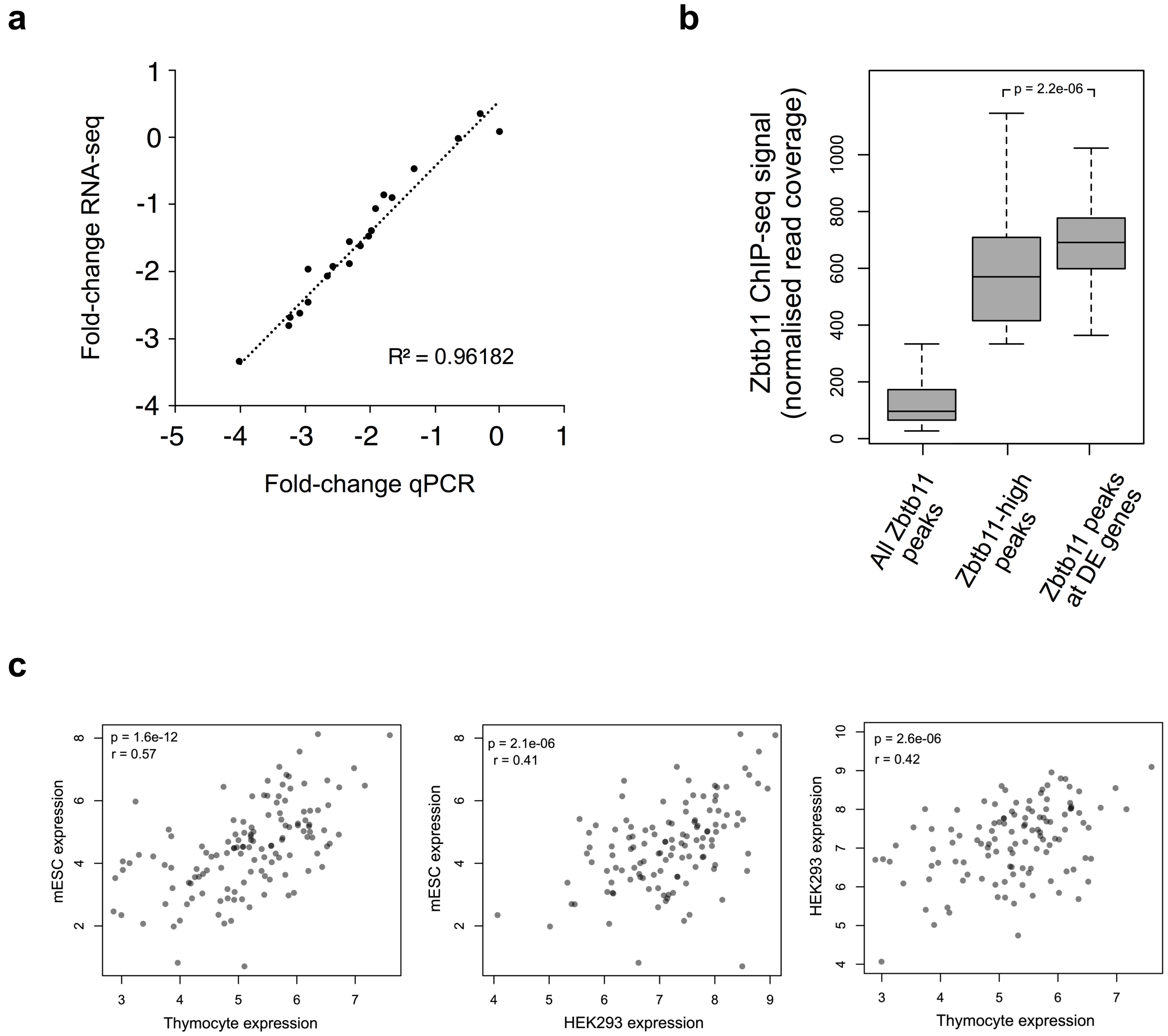

Supplementary Fig. 3

Supplementary Fig. 3

(a) Fold-change values obtained by RNA-seq for 20 genes plotted against fold-change values obtained by qRT-PCR for the same genes. qRT-PCR values represent mean of n=3 (*Ankrd40, Hus1, Mrpl1, Mrpl3, Mrpl30, Mrps31, Mrpl48, Ndufc2, Slc35a3, Taco1, Trdmt1*) and n=5 (*Acsf3*, *Bbc3*, *Ict1, Mrpl13, Mrpl16, Mrpl44, Mtg1, Ndufaf1, Trp53*) independent experiments.

(b) Boxplot showing the distribution of Zbtb11 ChIP-seq signal strength among peaks found at promoters of DE genes, compared to Zbtb11-high peaks, and the entire peak set. Outlier values in the distribution of the entire Zbtb11 peak set are not shown as these make up the Zbtb11-high group (see also Supplementary Fig.1f). P-value (Wilcoxon rank sum test) is shown above. Box plots show the interquartile range (box outline) and median value (horizontal line), with the whiskers delineating the lower and upper limits of the data. Note that peaks found at promoters of DE genes are overall significantly stronger than the rest.

(c) Correlation of transcript abundance for Zbtb11-dependent genes across different cell types and species. The Pearson correlation coefficients (r) and p-values for two-sided correlation tests (p) are shown.

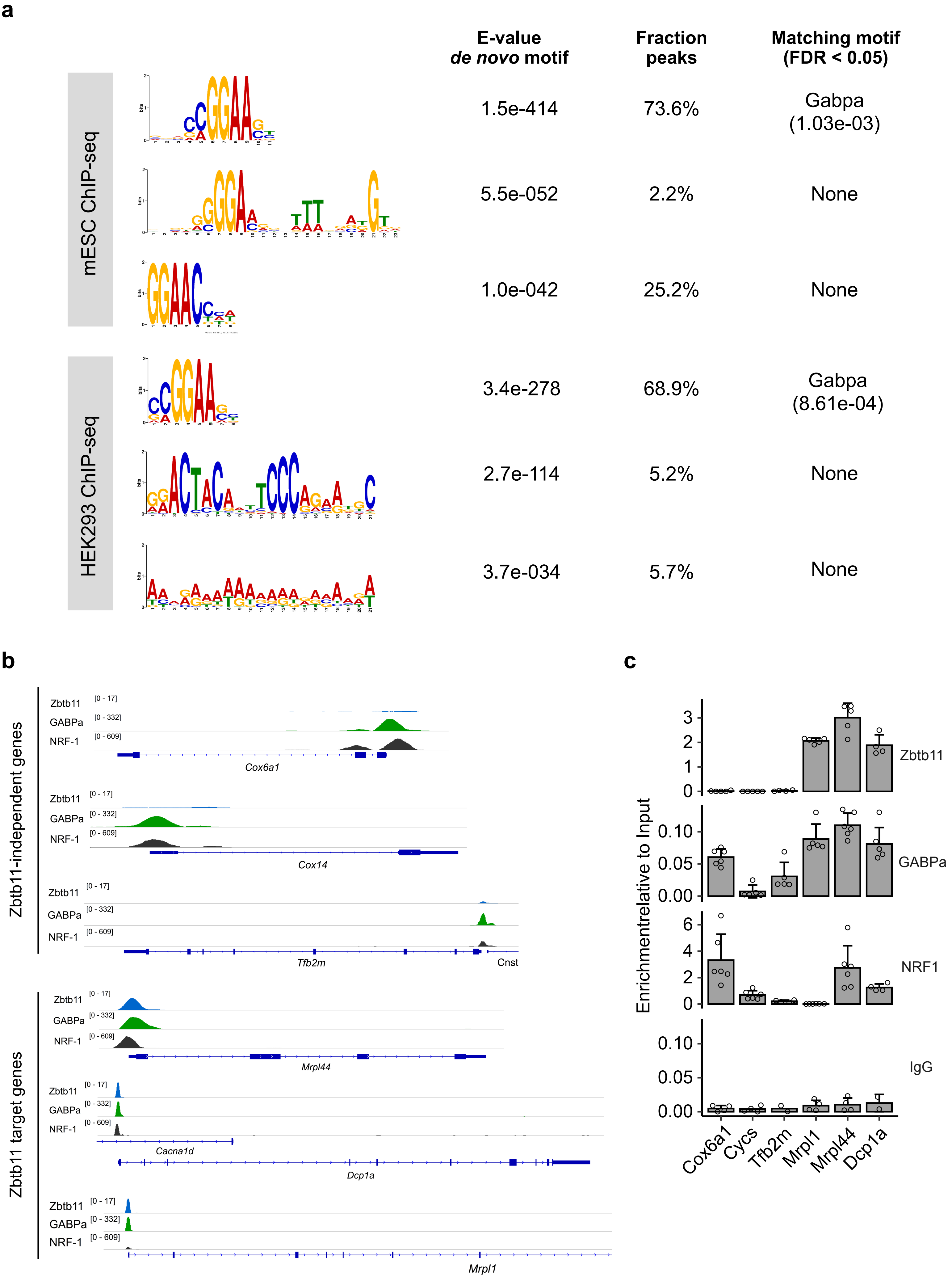

Supplementary Fig. 4

Supplementary Fig. 4

(a) Results of *de novo* motifs analyses using the sequences of Zbtb11-high ChIP-seq peaks identified in mouse ESCs and human HEK293 cells, respectively.

(b) Read coverage tracks for Zbtb11, GABPa and NRF-1 ChIP-seq in WT mouse ESCs. Three Zbtb11-independent loci (top) and three Zbtb11-dependent loci (bottom) are shown.

(c) ChIP-qPCR for Zbtb11, GABPa, NRF-1 and control rabbit IgG. Bars are mean and SD of enrichment values relative to Input detected with primers amplifying the promoter regions of the genes indicated underneath.

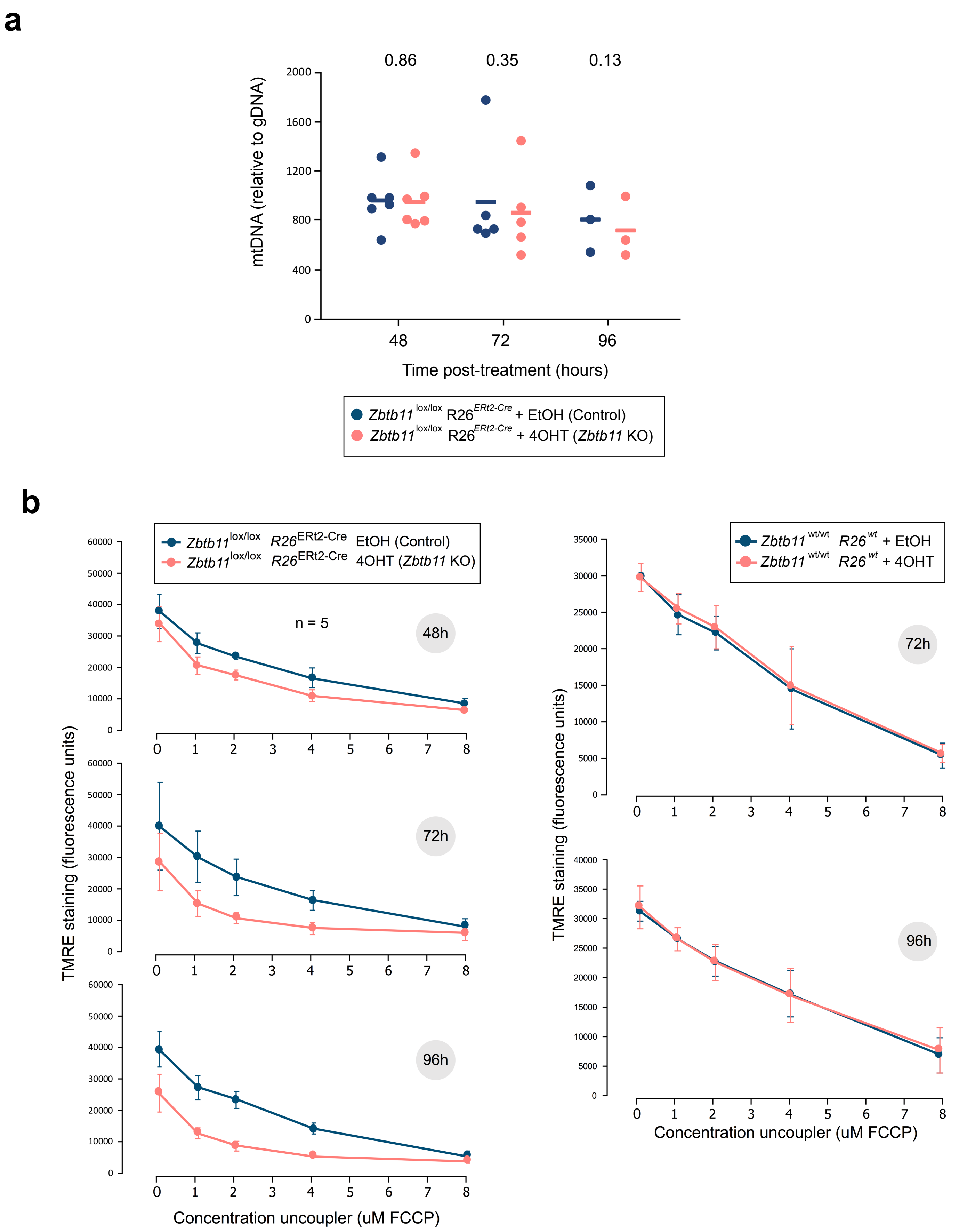

Supplementary Fig. 5

Supplementary Fig. 5

(a) qPCR quantification of mtDNA content relative to gDNA following *Zbtb11* KO induction. Individual measurements and means are shown for n=6 (48h), n=5 (72h) and n=3 (96h) independent experiments.

(b) Increased sensitivity of the MMP to mitochondrial uncoupler treatment in *Zbtb11* KO cells. Panels to the left - control and *Zbtb11* KO cells (time post-4OHT treatment indicated on the right) were stained with TMRE in the presence of increasing amounts of FCCP, and analysed by flow cytometry. Plots show TMRE mean fluorescence intensity against FCCP concentration (mean ± SD, n = 4-7 biological replicates). Panels to the right - to control for potential non-specific effects of the 4OHT treatment on the MMP, the TMRE staining and FCCP titration was carried out in EtOH- and 4OHT-treated *Zbtb11*$^{wt/wt}$ *Rosa26*^{wt} cells, showing that 4OHT treatment of wild type cells does not affect the MMP.

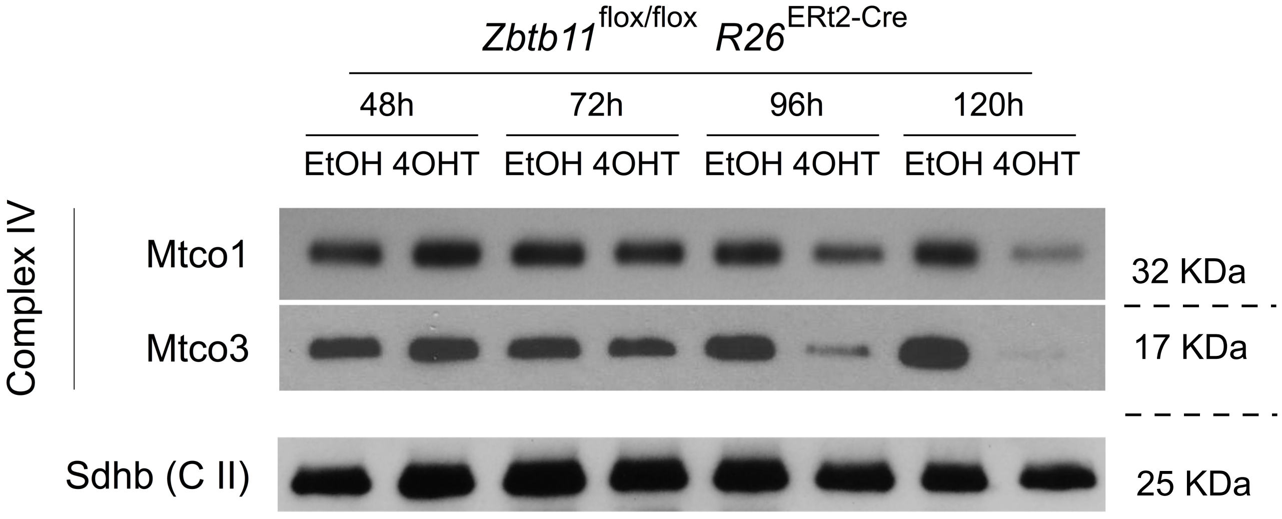

Supplementary Fig. 6

Supplementary Fig. 6

SDS-PAGE of mitochondrial extracts from EtOH-treated (control) and 4OHT-treated (*Zbtb11* KO) *Zbtb11*$^{lox/lox}$ *Rosa26*$^{ERt2\text{-}Cre}$ cells, followed by immunoblotting showing the mitochondria-encoded proteins Mtco1 and Mtco3 are down-regulated from 96 hours post-KO induction onwards. The nuclear-encoded complex II subunit Sdhb is shown as loading reference. Similar results were obtained for two independent experiments. Source data are provided as a Source Data file.

a

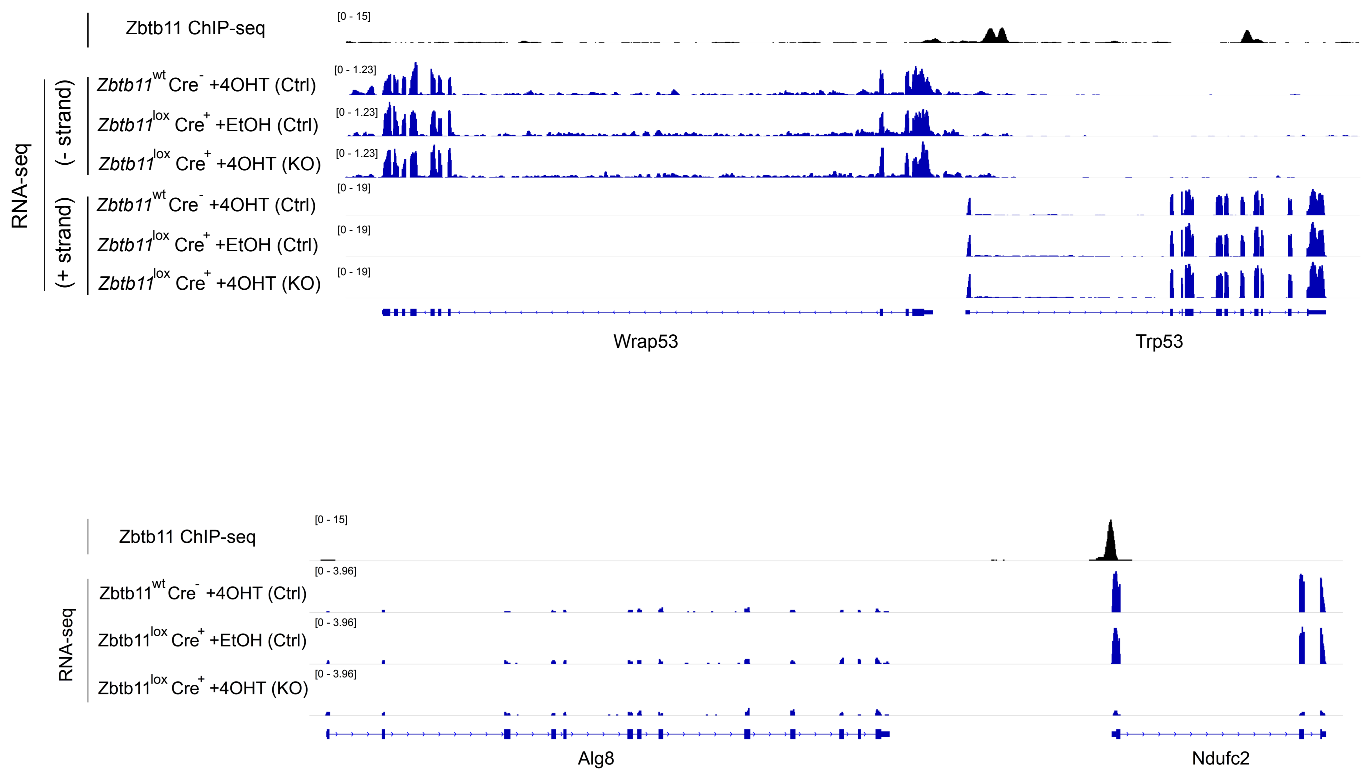

b

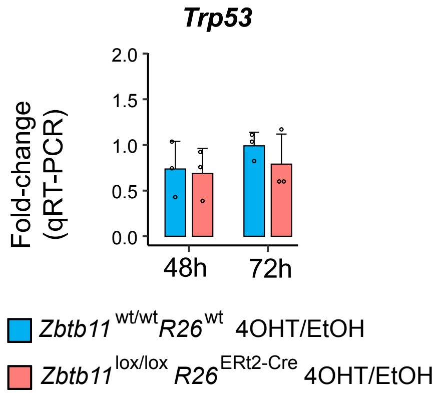

Supplementary Fig. 7

Supplementary Fig. 7

(a) Visualisation of normalised read coverage tracks for Zbtb11 ChIP-seq in control cells, and for RNA-seq in control and *Zbtb11* KO cells. Top panel – locus encoding p53 (*Trp53*) showing no transcriptional changes. Bottom panel - *Ndufc2* locus shown for comparison, illustrating transcriptional down-regulation.

(b) qRT-PCR analysis of *Trp53* transcription in control and *Zbtb11* KO cells, 48 and 72 hours post-KO induction. Bars show mean and SD of 3-5 biological replicates.

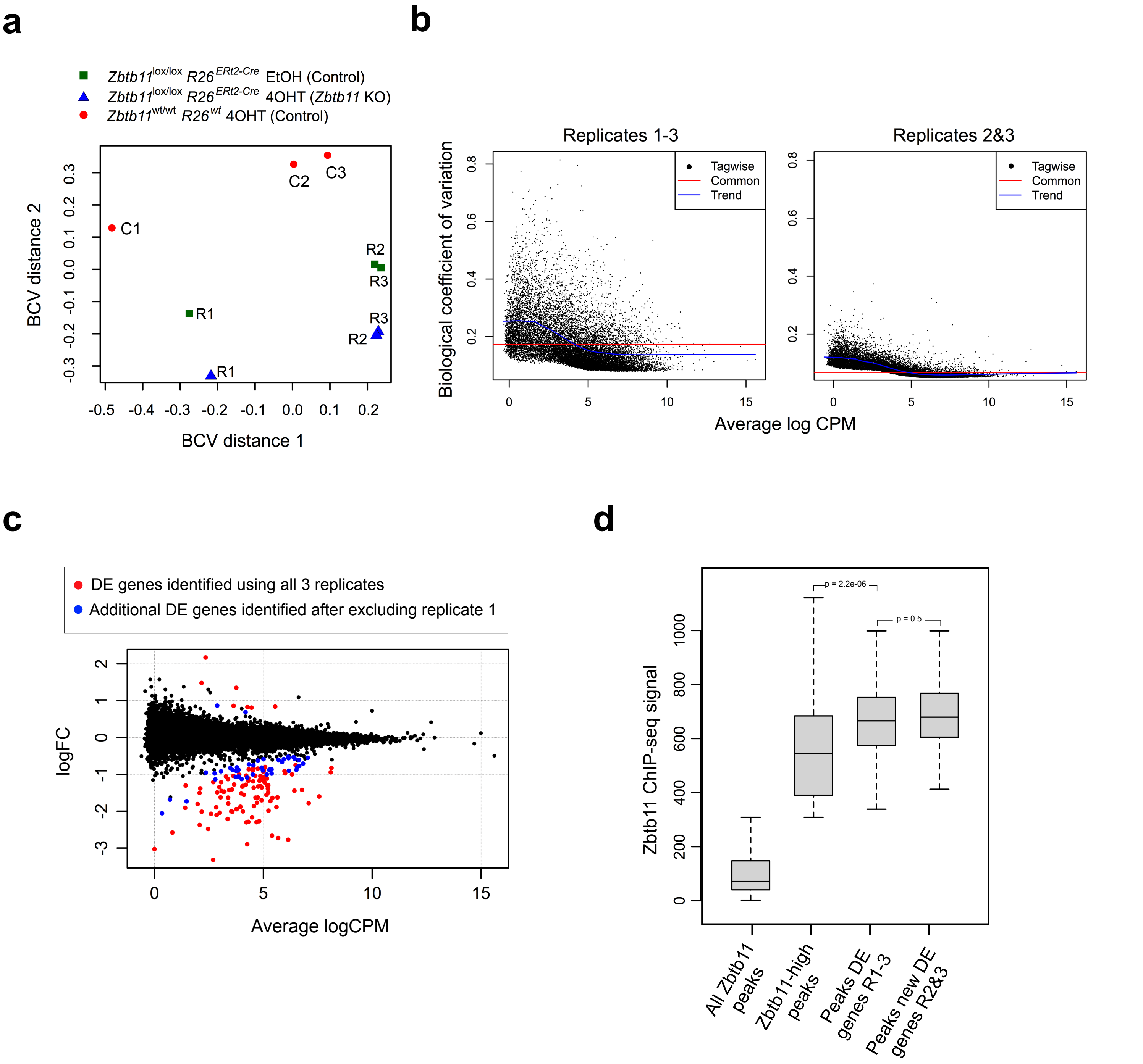

Supplementary Fig. 8

Supplementary Fig. 8

(a) Multidimensional scaling (MDS) plot of the RNA-seq libraries.

(b) Biological coefficient of variation (BCV) plotted against transcript abundance (mean counts per million) when all three RNA-seq replicates are included in the analysis (left) or when replicate 1 is removed (right).

(c) Plot of log2-transformed fold-change against transcript abundance obtained after removing RNA-seq replicate 1 from the differential gene expression analysis. In red are shown DE genes found by using all three replicates, while additional DE genes identified after removal of replicate 1 are shown in blue.

(d) Boxplot of Zbtb11 ChIP-seq signal showing that peaks at promoters of the additional DE genes identified once RNA-seq replicate 1 is removed are as strong as the peaks found at the DE genes initially identified with all three RNA-seq replicates. P-values for pair-wise comparisons of the distributions are shown above the plot and were obtained by performing a two-sided Wilcoxon rank sum test. Box plots show the interquartile range (box outline) and median value (horizontal line), with the whiskers delineating the lower and upper limits of the data.

Flow cytometry gating strategy for measuring AnnexinV-positive cells in Figure 2e and Supplementary Figure 2d.

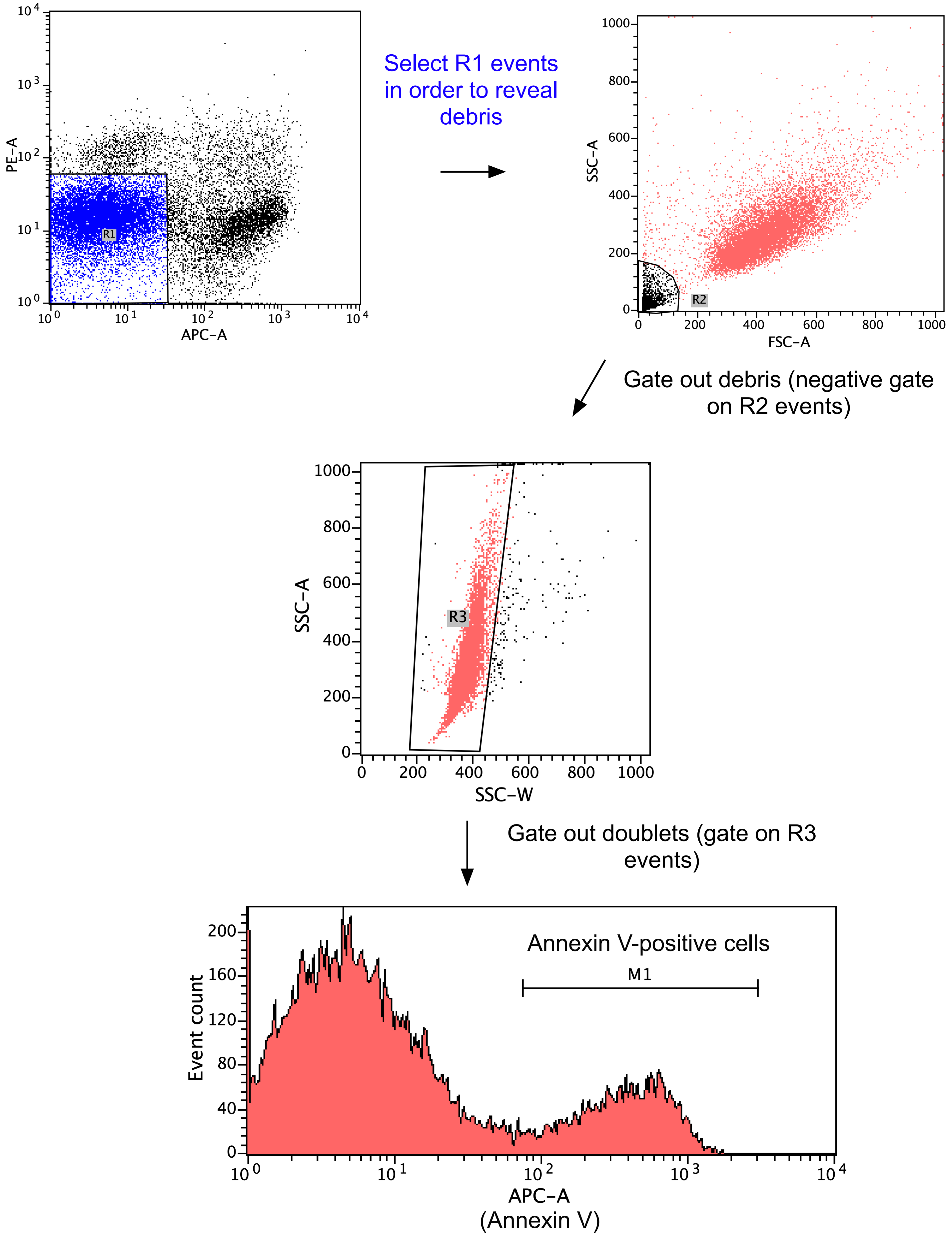

Supplementary Fig. 9

Supplementary Fig. 9

Flow cytometry gating strategy for measuring AnnexinV-positive cells in Figure 2e and Supplementary Figure 2d.

Supplementary Fig. 10. Uncropped immunoblots used in the figures.

Fig. 1b

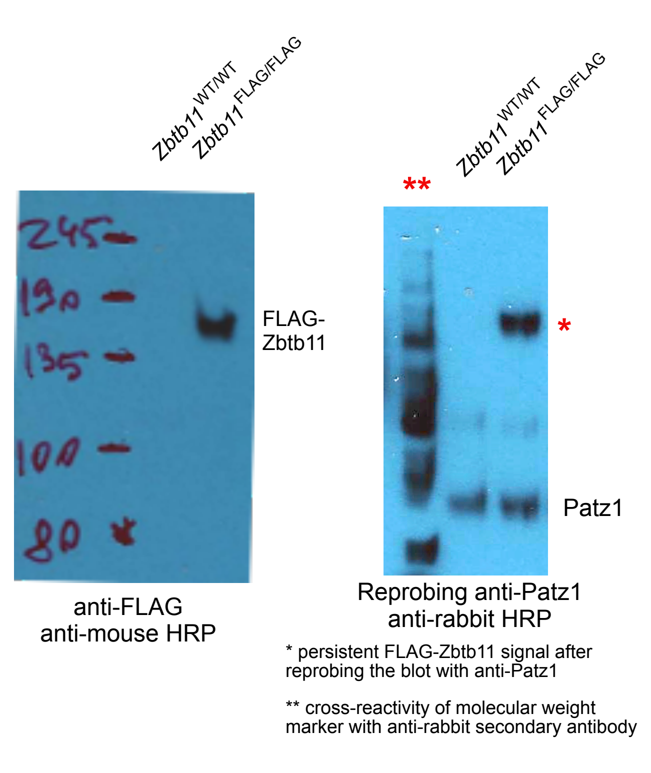

Fig. 3b

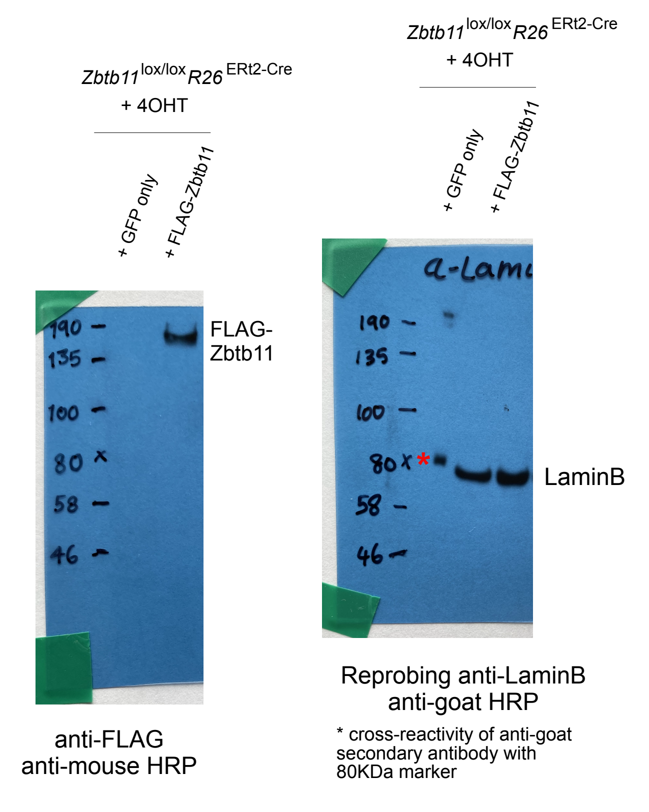

Fig. 6b

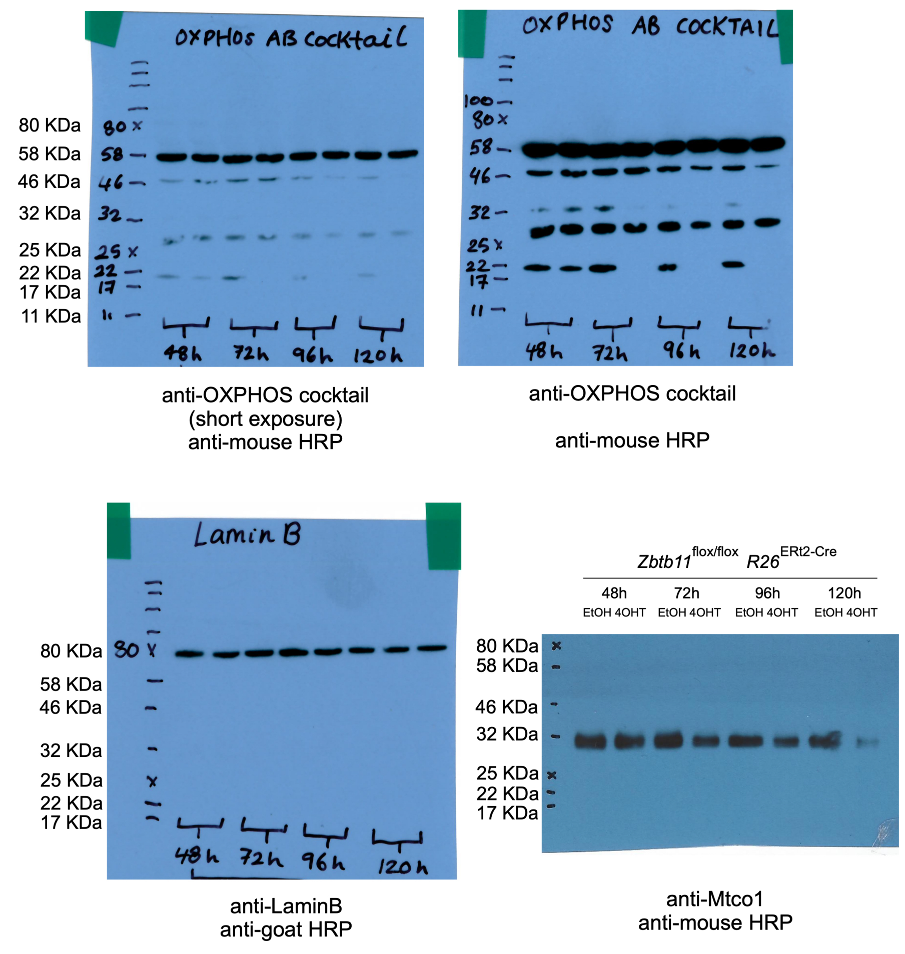

Fig. 6d

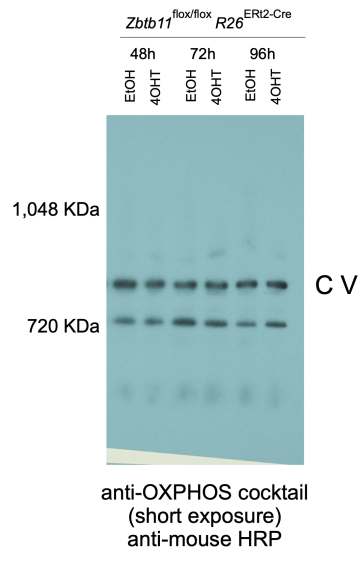

Fig. 6c

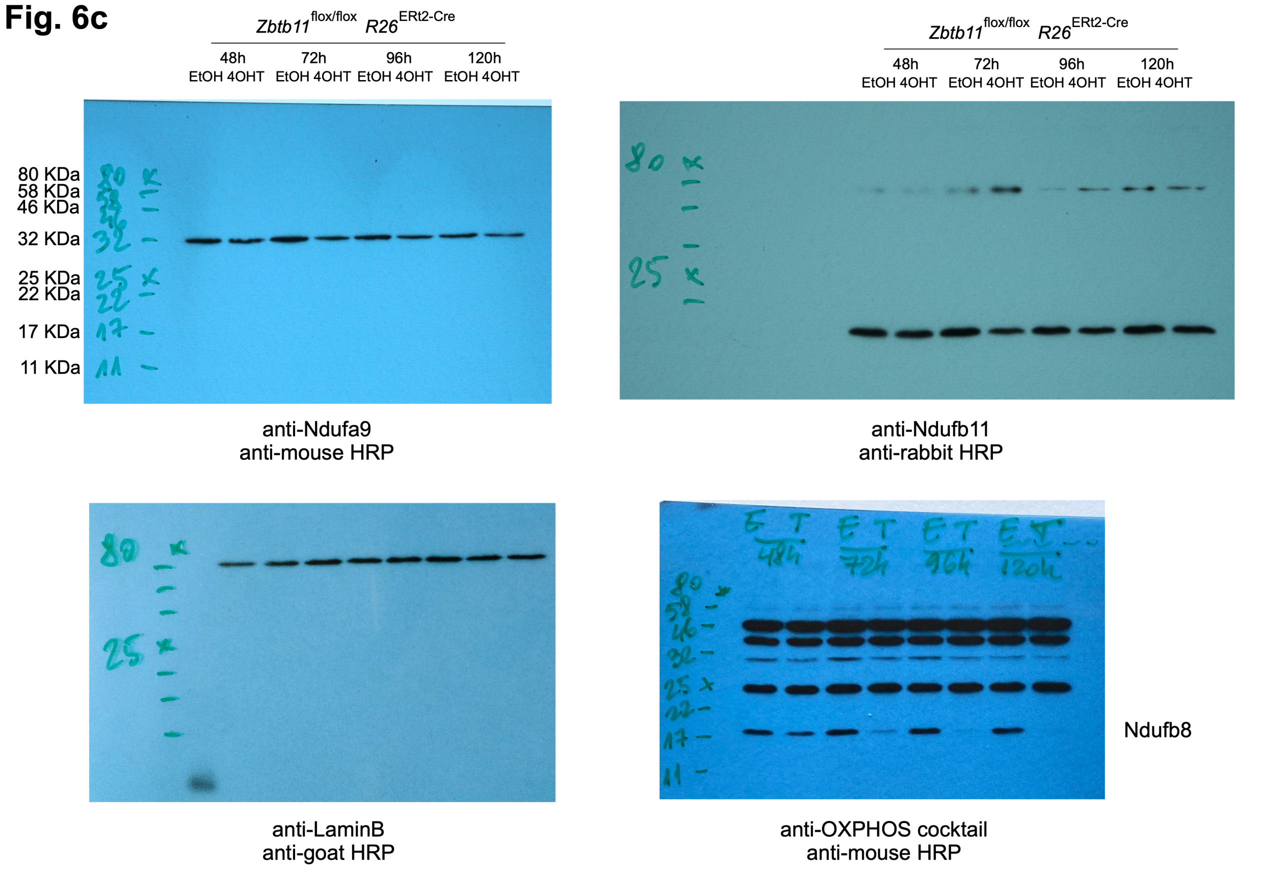

Fig. 7a

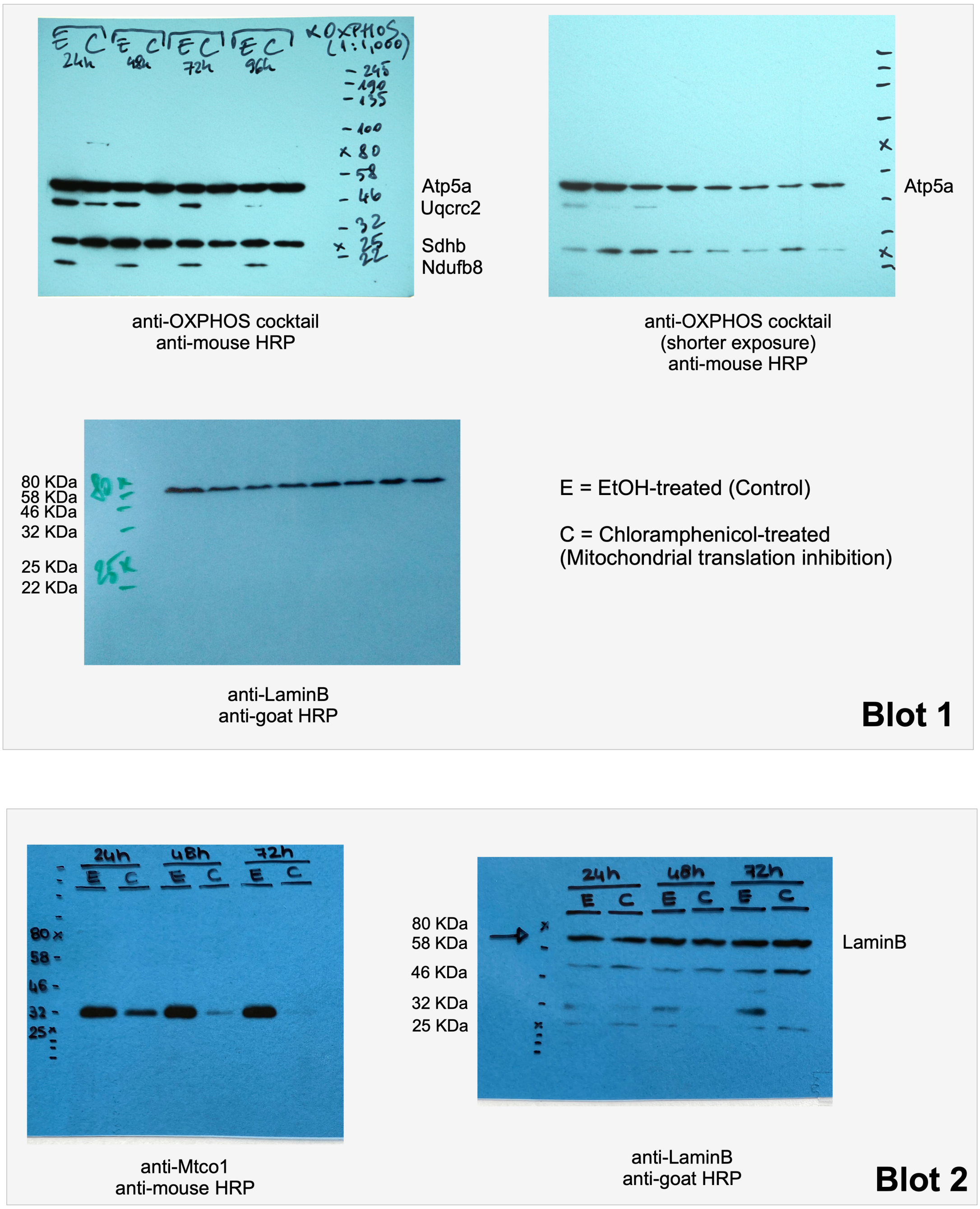
Atp5a
Uqcrc2
Sdhb
Ndufb8
anti-OXPHOS cocktail
anti-mouse HRP
Atp5a
anti-OXPHOS cocktail
(shorter exposure)
anti-mouse HRP
80 KDa
58 KDa
46 KDa
32 KDa
25 KDa
22 KDa
anti-LaminB
anti-goat HRP
E = EtOH-treated (Control)
C = Chloramphenicol-treated
(Mitochondrial translation inhibition)
Blot 1
24h
48h
72h
E
C
E
C
E
C
anti-Mtco1
anti-mouse HRP
80 KDa
58 KDa
46 KDa
32 KDa
25 KDa
LaminB
anti-LaminB
anti-goat HRP
Blot 2

Fig. 7c - left panel

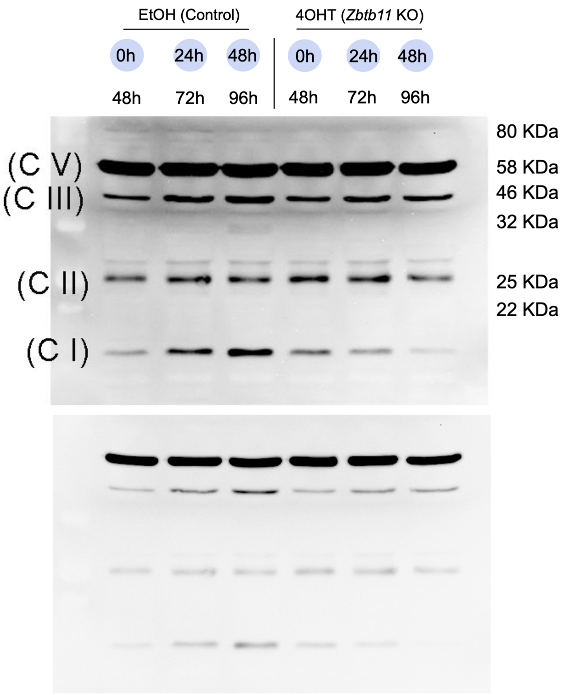

anti-OXPHOS cocktail
anti-mouse HRP

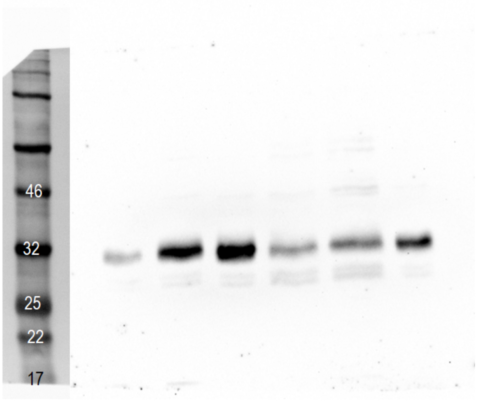

anti-Mtco1
anti-mouse HRP

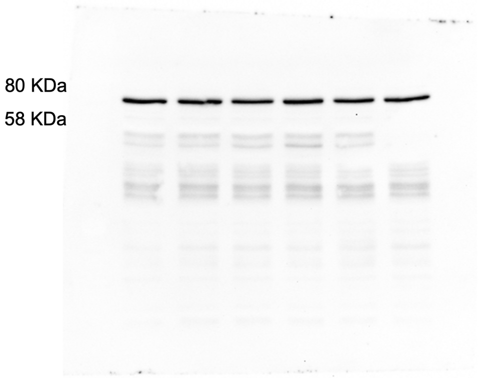

anti-LaminB
anti-goat HRP

Fig. 7c - right panel

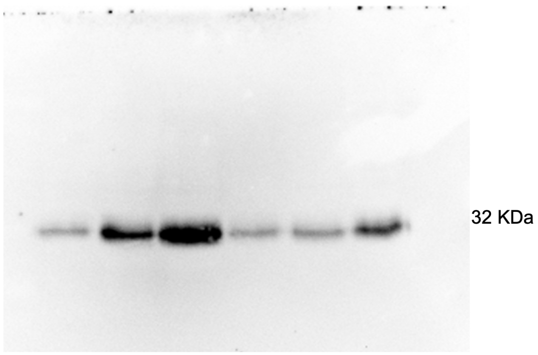

anti-Mtco1
anti-mouse HRP

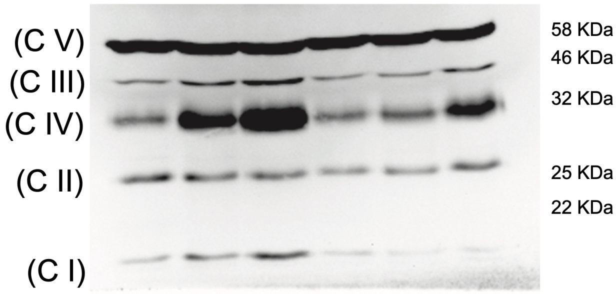

anti-OXPHOS cocktail
anti-mouse HRP

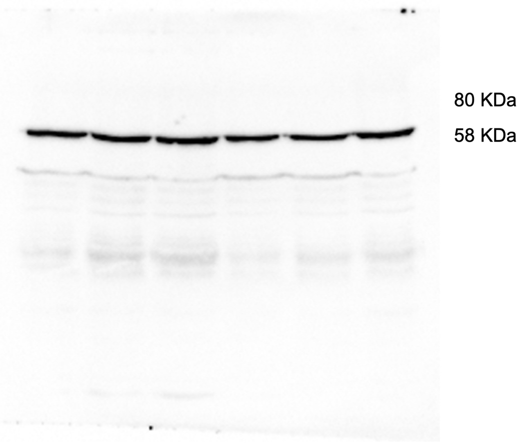

anti-LaminB
anti-goat HRP

Fig. 8b

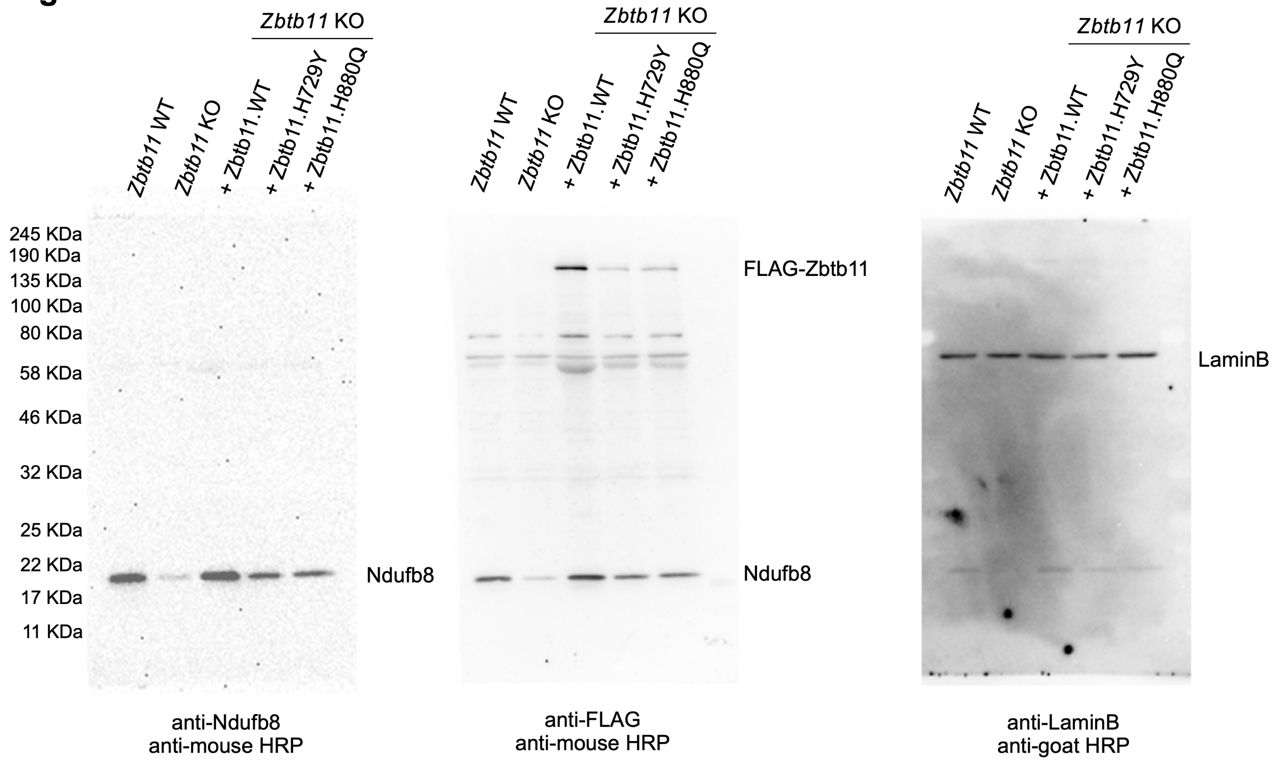
Zbtb11 KO
Zbtb11 WT
Zbtb11 KO
+ Zbtb11.WT
+ Zbtb11.H729Y
+ Zbtb11.H880Q
245 KDa
190 KDa
135 KDa
100 KDa
80 KDa
58 KDa
46 KDa
32 KDa
25 KDa
22 KDa
17 KDa
11 KDa
Ndufb8
anti-Ndufb8
anti-mouse HRP
FLAG-Zbtb11
Ndufb8
anti-FLAG
anti-mouse HRP
LaminB
anti-LaminB
anti-goat HRP

Supplementary Fig. 1a

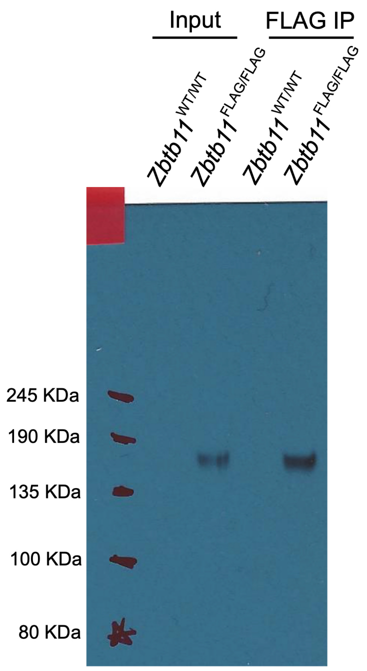

anti-FLAG
anti-mouse HRP

Supplementary Fig. 6

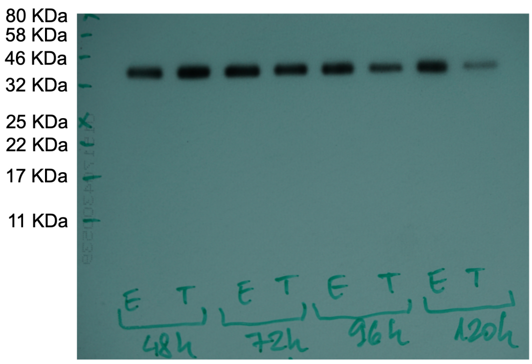

anti-Mtco1
anti-mouse HRP

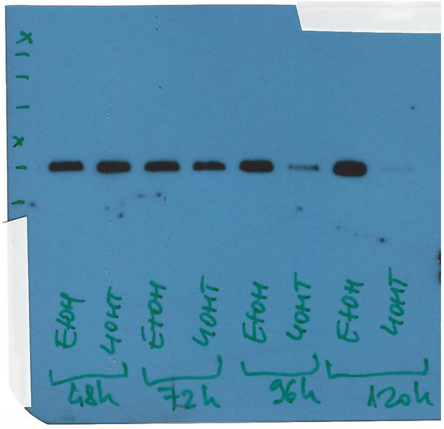

anti-Mtco3
anti-mouse HRP

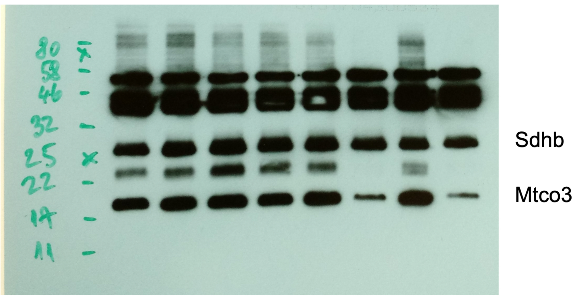

anti-OXPHOS cocktail
anti-mouse HRP
