# Supplementary material for: Intellectual disability-associated factor Zbtb11 cooperates with NRF-2/GABP to control mitochondrial function
Source: Nat Commun. 2020 Oct 29;11:5469. doi: 10.1038/s41467-020-19205-x (PMC7596099; doi:10.1038/s41467-020-19205-x)
Supplement: Supplementary file 4 — Reporting Summary [file 41467_2020_19205_MOESM4_ESM.pdf]

nature research

Corresponding author(s): Vlad Seitan

Last updated by author(s): Jan 14, 2020

Reporting Summary

Nature Research wishes to improve the reproducibility of the work that we publish. This form provides structure for consistency and transparency in reporting. For further information on Nature Research policies, see Authors & Referees and the Editorial Policy Checklist.

Statistics

For all statistical analyses, confirm that the following items are present in the figure legend, table legend, main text, or Methods section.

n/a	Confirmed	
☐	☒	The exact sample size (*n*) for each experimental group/condition, given as a discrete number and unit of measurement
☐	☒	A statement on whether measurements were taken from distinct samples or whether the same sample was measured repeatedly
☐	☒	The statistical test(s) used AND whether they are one- or two-sided<br>*Only common tests should be described solely by name; describe more complex techniques in the Methods section.*
☒	☐	A description of all covariates tested
☐	☒	A description of any assumptions or corrections, such as tests of normality and adjustment for multiple comparisons
☐	☒	A full description of the statistical parameters including central tendency (e.g. means) or other basic estimates (e.g. regression coefficient) AND variation (e.g. standard deviation) or associated estimates of uncertainty (e.g. confidence intervals)
☐	☒	For null hypothesis testing, the test statistic (e.g. *F*, *t*, *r*) with confidence intervals, effect sizes, degrees of freedom and *P* value noted<br>*Give P values as exact values whenever suitable.*
☒	☐	For Bayesian analysis, information on the choice of priors and Markov chain Monte Carlo settings
☒	☐	For hierarchical and complex designs, identification of the appropriate level for tests and full reporting of outcomes
☐	☒	Estimates of effect sizes (e.g. Cohen's *d*, Pearson's *r*), indicating how they were calculated

Our web collection on statistics for biologists contains articles on many of the points above.

Software and code

Policy information about availability of computer code

Data collection	Oxygen concentrations/consumption was collected on a Seahorse XF24 running XFReader (version 1.8.1.1)<br>qPCR data was collected using a StepOne Plus system (Applied Biosystems) running StepOne Software (version 2.3)<br>Protein concentration measurements were collected using a plate reader running SkanIt Software (version 5.0)
Data analysis	RNA-seq reads were aligned using STAR (version 2.5.1a)<br>Differential gene expression analysis was performed in R using the Bioconductor package edgeR (version 3.24.0)<br>ChIP-seq reads were trimmed and filtered using Trim Galore!(Cutadapt) (version 0.4.3), and duplicates removed with Picard (version 2.6.0). Filtered reads were aligned using Bowtie (version 1.1.2).<br>Chip-seq peaks were called using MACS2 (version 2.1.0) and consensus peaksets were obtained using the Irreproducibility Discovery Rate pipeline (version 2012) as described: https://sites.google.com/site/anshulkundaje/projects/idr/deprecated<br>Overlap analysis of genomic regions was performed using the R Bioconductor packages GenomicRanges (version 1.36.1) and GenomicFeatures (version 1.36.4). Normalised read coverage values for consensus peaks were obtained using the DiffBind Bioconductor package (version 2.12.0).<br>Specific genomic sequences were obtained using the Bioconductor package BSgenome (version 1.52.0).

For manuscripts utilizing custom algorithms or software that are central to the research but not yet described in published literature, software must be made available to editors/reviewers. We strongly encourage code deposition in a community repository (e.g. GitHub). See the Nature Research guidelines for submitting code & software for further information.

Data

Policy information about availability of data

All manuscripts must include a data availability statement. This statement should provide the following information, where applicable:

- Accession codes, unique identifiers, or web links for publicly available datasets
- A list of figures that have associated raw data
- A description of any restrictions on data availability

The datasets generated during during the current study are available in the GEO repository, accession number GSE125047, https://www.ncbi.nlm.nih.gov/geo/query/acc.cgi?acc=GSE125047
Figures with associated raw data: Fig.1, Fig.3, Supplementary Fig. 1

Field-specific reporting

Please select the one below that is the best fit for your research. If you are not sure, read the appropriate sections before making your selection.

☒ Life sciences ☐ Behavioural & social sciences ☐ Ecological, evolutionary & environmental sciences

For a reference copy of the document with all sections, see nature.com/documents/nr-reporting-summary-flat.pdf

Life sciences study design

All studies must disclose on these points even when the disclosure is negative.

Sample size	The aim of this study was to identify the molecular and cellular roles of Zbtb11. We used a genetic approach to delete the Zbtb11 gene and given that very little is known about this transcription factor, we were interested in identifying the most striking phenotypes. We were therefore interested in relatively large effect sizes (> 2-fold changes between control and KO). Given we have carried out the assays before, we knew the expected signal to noise ratio. This allowed us to conduct power calculations to estimate sample sizes that provide 80% chance of detecting a significant 2-fold difference between control and Zbtb11 KO when a p-value cutoff of 0.05 is applied. The formula used to determine expected signal to noise ratio is (signal/noise ratio) = (Mean KO -Mean Control)/standard deviation. Tables providing necessary sample sizes for discrete values of signal/noise ratios and power are widely available in statistical books. An example: http://www.3rs-reduction.co.uk/html/6__power_and_sample_size.html<br>We do however recognise there are limitations in applying null hypothesis significance testing, especially where sample sizes are small, and that p-values are better considered as continuous values. For this reason we provide in the figures actual p-values rather than designations of significance.
Data exclusions	One RNA-seq biological replicate was excluded from the differential gene expression (DGE) analysis, based on the results of multi-dimensional scaling (MDS) analysis which showed that one replicate was considerably different from the other two. The full justification for this and a comparison of the results with and without the excluded replicate are included in the Methods section and Supplementary Fig. 7
Replication	As much as possible we tried to validate our findings by using more than one approach to measure differences between control and Zbtb11 KO samples. For example, RNA-seq results were validated by performing RT-qPCR on independent samples. Similarly, binding of Zbtb11 to the chromatin was investigated by ChIP-seq as well as ChIP-qPCR. Importantly, the binding of Zbtb11 to the chromatin was determined using antibodies to the endogenous protein as well as FLAG antibodies in a cell line expressing FLAG-Zbtb11, obtaining highly correlated datasets.
Randomization	We obtained control and Zbtb11 KO samples by splitting the same culture in two and applying either ethanol (carrier) or 4-hydroxytamoxyfen (4OHT) treatment, so there should be no confounding covariates. The effect of 4OHT alone was controlled for by including 4OHT-treated Zbtb11 WT cells in the experimental design.
Blinding	Where possible, samples were collected and analysed by different researchers. Due to limitations on staff working on the same project this was not always possible.

Reporting for specific materials, systems and methods

We require information from authors about some types of materials, experimental systems and methods used in many studies. Here, indicate whether each material, system or method listed is relevant to your study. If you are not sure if a list item applies to your research, read the appropriate section before selecting a response.

Materials & experimental systems

n/a	Involved in the study
☐	☒ Antibodies
☐	☒ Eukaryotic cell lines
☒	☐ Palaeontology
☒	☐ Animals and other organisms
☒	☐ Human research participants
☒	☐ Clinical data

Methods

n/a	Involved in the study
☐	☒ ChIP-seq
☐	☒ Flow cytometry
☒	☐ MRI-based neuroimaging

Antibodies

Antibodies used

FLAG M2 (F3165, Sigma)
Zbtb11 (NBP1-80327, Bio-Techne)
GABPa (sc-28312 X, Santa Cruz)
NRF1 (D9K6P) (46743S, Cell Signalling Technologies)
Patz1 (sc-292109, Santa Cruz)
Lamin B (sc-6216, Santa Cruz)
Mrpl48 (14677-1-AP, Proteintech)
Ndufc2 (15573-1-AP, Proteintech)
Ndufb11 (ab183716, Abcam)
Ndufa9 (ab14713, Abcam)
Mtco1, Ndufb8, Sdhb, Uqcrc2, and Atp5a are part of an OXPHOS antibody cocktail (ab110413, Abcam)
Mtco1 (ab14705, Abcam)
HRP-coupled anti-mouse (P026002-2, Dako)
HRP-coupled anti-rabbit (A16029, Thermo Fisher Scientific)
HRP-coupled anti-goat (SAB3700285, Sigma)
Alexa Fluor 647-conjugated anti-cleaved Caspase-3 (9602, Cell Signaling Technology)

Validation

Zbtb11 (NBP1-80327, Bio-Techne) and Patz1 (sc-292109, Santa Cruz) were validated by loss of signal in the respective KO cells (see Figures 2b and Supplementary Figure 2b).
Zbtb11 (NBP1-80327, Bio-Techne) ChIP-seq signal also correlated very well with FLAG-Zbtb11 ChIP-seq (See Figure 1c and Supplementary Figure 1c).

The rest of the antibodies were extensively characterised in the literature by immunoblot:
FLAG M2 (F3165, Sigma) - The Journal of biological chemistry, 279(53), 55697-55706 (2004), Jean-Christophe Peter et. al,
GABPa (sc-28312 X, Santa Cruz) - Cell Stem Cell. 23: 193-209.e5. , 2018, Yu, B. et al.
Patz1 (sc-292109, Santa Cruz) - Molecular and Cellular Biology,2015, Keskin, N., Deniz, E., et al.
NRF1 (D9K6P) (46743S, Cell Signalling Technologies) - Cell Discovery, 2018, Liao, C. Y., Anderson, S. S., et al.
Mrpl48 (14677-1-AP, Proteintech) - Nucleic Acids Research, 2013, Surovtseva, Y. V. & Shadel, G. S..
Ndufc2 (15573-1-AP, Proteintech) - Scientific Reports, 2017, Fu, L., Xu, Y., et al.
Ndufb11 (ab183716, Abcam) - Clinical Genetics, 2018 , Baertling, F., Sánchez-Caballero, L., et al.
Ndufa9 (ab14713, Abcam) - Cell Research, 2019, Rao, S., Mondragón, L., et al.
Mtco1, Ndufb8, Sdhb, Uqcrc2, and Atp5a are part of an OXPHOS antibody cocktail (ab110413, Abcam) - Nat Commun 9:1875 (2018), Lin H et al.
Mtco1 (ab14705, Abcam) - Nature,2018 , Frattini, V., Pagnotta, S. M., et al.
HRP-coupled anti-mouse (P026002-2, Dako) - Neuron. 2017;94(4):809-825.e7., Tortosa E et al
HRP-coupled anti-rabbit (A16029, Thermo Fisher Scientific) - Nature Neuroscience, 2015, Zhao, Z., Sagare, A. P., et al.
HRP-coupled anti-goat (SAB3700285, Sigma) - Scientific reports, 9(1), 2019, Anna Georges et. al
Alexa Fluor 647-conjugated anti-cleaved Caspase-3 (9602, Cell Signaling Technology) - Cell, 2017, Moretti, J., Roy, S., et al.

Eukaryotic cell lines

Policy information about cell lines

Cell line source(s)

Gift from the transgenics core facility (MRC London Institute of Medical Sciences): wild type E14 mouse ES cells.
Oakey Lab (King's College London): HEK293

Authentication

E14 mouse ES cells: Expression of pluripotency markers Oct4, Nanog, Sox2. Microscopy - colony formation and morphology.
HEK293: Microscopy - morphology

Mycoplasma contamination

All cells tested negative for Mycoplasma

Commonly misidentified lines
(See ICLAC register)

No commonly misidentified cell lines were used in this study

ChIP-seq

Data deposition

☒ Confirm that both raw and final processed data have been deposited in a public database such as GEO.

☒ Confirm that you have deposited or provided access to graph files (e.g. BED files) for the called peaks.

Data access links
May remain private before publication.

https://www.ncbi.nlm.nih.gov/geo/query/acc.cgi?acc=GSE125047

Files in database submission

FLAG-Zbtb11_peaks.bed
FLAG_Control_peaks.bed
Zbtb11_peaks.bed
Zbtb11_consensus_peaks_read_coverage.txt
FLAG-Zbtb11_ChIPSeq_Rep1.bw.gz
FLAG-Zbtb11_ChIPSeq_Rep2.bw.gz
FLAG-Zbtb11_ChIPSeq_Rep3.bw.gz
FLAG_Control_ChIPSeq_Rep1.bw.gz
FLAG_Control_ChIPSeq_Rep2.bw.gz
Zbtb11_ChIPSeq_Rep1.bw.gz
Zbtb11_ChIPSeq_Rep2.bw.gz
Zbtb11_ChIPSeq_Rep3.bw.gz
Zbtb11_ChIPSeq_Rep4.bw.gz
Input_FLAG-Zbtb11_Rep1.bw.gz
Input_FLAG-Zbtb11_Rep2.bw.gz
Input_Zbtb11_Rep1.bw.gz
Input_Zbtb11_Rep2.bw.gz
Input_Zbtb11_Rep3.bw.gz
FLAG-Zbtb11_ChIPSeq_Rep1.fastq.gz
FLAG-Zbtb11_ChIPSeq_Rep2.fastq.gz
FLAG-Zbtb11_ChIPSeq_Rep3.fastq.gz
FLAG_Control_ChIPSeq_Rep1.fastq.gz
FLAG_Control_ChIPSeq_Rep2.fastq.gz
Zbtb11_ChIPSeq_Rep1.fastq.gz
Zbtb11_ChIPSeq_Rep2.fastq.gz
Zbtb11_ChIPSeq_Rep3.fastq.gz
Zbtb11_ChIPSeq_Rep4.fastq.gz
Input_FLAG-Zbtb11_Rep1.fastq.gz
Input_FLAG-Zbtb11_Rep2.fastq.gz
Input_Zbtb11_Rep1.fastq.gz
Input_Zbtb11_Rep2.fastq.gz
Input_Zbtb11_Rep3.fastq.gz

Genome browser session
(e.g. UCSC)

https://genome.ucsc.edu/s/Vladseitan/WilsonB_SeitanVC_Jan2020

Methodology

Replicates

Zbtb11_ChIPSeq 4 biological replicates Pairwise correlation coefficients: 0.8, 0.83, 0.77, 0.81, 0.78, 0.8
FLAG-Zbtb11_ChIPSeq 3 biological replicates Pairwise correlation coefficients: 0.83, 0.82, 0.77
FLAG_Control_ChIPSeq 2 biological replicates Pairwise correlation coefficients: N/A (control samples)

Sequencing depth

SAMPLE	TOTAL READS	UNIQUELY MAPPED READS
FLAG-Zbtb11_ChIPSeq_Rep1	162,113,122	107,834,690
FLAG-Zbtb11_ChIPSeq_Rep2	111,185,522	76,050,172
FLAG-Zbtb11_ChIPSeq_Rep3	34,637,440	23,839,146
FLAG_Control_ChIPSeq_Rep1	138,627,144	91,697,962
FLAG_Control_ChIPSeq_Rep2	73,286,570	49,200,154
Zbtb11_ChIPSeq_Rep1	91,510,697	63,113,855
Zbtb11_ChIPSeq_Rep2	94,100,548	62,993,772
Zbtb11_ChIPSeq_Rep3	31,320,381	22,260,708
Zbtb11_ChIPSeq_Rep4	32,833,640	23,107,422

Antibodies

FLAG M2 (F3165, Sigma)
Zbtb11 (NBP1-80327, Bio-Techne)

Peak calling parameters

Read mapping (Bowtie [1.1.2]): bowtie -p 4 -S -v 2 -m 1
Reads were aligned to mm9

Peak calling (MACS2 [2.1.0]) - part of the Irreproducibility Discovery Rate (IDR) pipeline which requires calling > 100,000 peaks with a relaxed threshold of p-value 0.05 or 0.1 (macs2 callpeak -p 0.05) in order to provide a suitable background for

the IDR calculations. The IDR threshold used was 0.02 and the final set of peaks was the conservative peakset.
For peak calling PCR/optical duplicates were removed, and peaks were called relative to control (input) samples.

Data quality

We used an Irreproducibility Discovery Rate (IDR) threshold of 0.02 (conservative peakset). This approach has been used by the ENCODE consortium processing pipeline and is described in detail:
https://sites.google.com/site/anshulkundaje/projects/idr/deprecated

SAMPLE	NUMBER OF PEAKS
Zbtb11_ChIPSeq (IDR 4 replicates)	8,957
FLAG-Zbtb11_ChIPSeq (IDR 3 replicates)	9,350
FLAG_Control_ChIPSeq (IDR 2 replicates)	327
Consensus Zbtb11/FLAG-Zbtb11	7,500

Software

Peaksets determined by IDR (BED) and alignment files (BAM) were imported in R using the Bioconductor package DiffBind and normalised read coverage values were obtained for each peak. Peaks were annotated using the Bioconductor package TxDb.Mmusculus.UCSC.mm9.knownGene

Flow Cytometry

Plots

Confirm that:

☒ The axis labels state the marker and fluorochrome used (e.g. CD4-FITC).

☒ The axis scales are clearly visible. Include numbers along axes only for bottom left plot of group (a 'group' is an analysis of identical markers).

☒ All plots are contour plots with outliers or pseudocolor plots.

☒ A numerical value for number of cells or percentage (with statistics) is provided.

Methodology

Sample preparation

ES cells were fixed in 70% ethanol and stained with propidium iodide for cell cycle analysis
For cell GFP-based sorting, cells were trypsinised, washed in PBS and resuspended in PBS supplemented with fetal bovine serum and DAPI to discriminate dead cells

Instrument

BD LSRFortessa for analysis
BD FACSAria for cell sorting

Software

BS FACSDiva

Cell population abundance

The sort target for each experiment was set at 100,000 cells per sample. Samples were always sorted sequentially on the same machine and using identical settings for all samples in one experiment. Purity was determined by analysing a small fraction of cells from the sorted samples using the same cytometer used for the sort.

Gating strategy

Cell debris were gated out on a SSC-A/FSC-A plot, doublets were then removed on a SSC-A/SSC-W plot, and live cells were selected on a Pacific Blue-A/FSC-A plot by gating out DAPI-positive cells. Gated live cells were then visualised on a PE-A/FITC-A plot in order to discriminate true GFP-positive cells from autofluoresce.

☒ Tick this box to confirm that a figure exemplifying the gating strategy is provided in the Supplementary Information.
